# Tackling dementia globally: the Global Dementia Prevention Program (GloDePP) collaboration

**DOI:** 10.7189/jogh.09.020103

**Published:** 2019-12

**Authors:** Kit Yee Chan, Davies Adeloye, Kwaku Poku Asante, Clara Calia, Harry Campbell, Samuel O Danso, Sanjay Juvekar, Saturnino Luz, Devi Mohan, Graciela Muniz-Terrera, Ricardo Nitrini, Maryam Noroozian, Amit Nulkar, Solomon Nyame, Vasudeo Paralikar, Mario A Parra Rodriguez, Adrienne N. Poon, Daniel D Reidpath, Igor Rudan, Blossom CM Stephan, TinTin Su, Huali Wang, Tam Watermeyer, Heather Wilkinson, Monica Sanches Yassuda, Xin Yu, Craig Ritchie, on behalf of the Global Dementia Prevention Program (GloDePP)

**Affiliations:** 1 Centre for Global Health, Usher Institute, University of Edinburgh, United Kingdom; 2 Nossal Institute for Global Health, Melbourne School of Population and Global Health, University of Melbourne, Australia; 3 RcDavies Evidence-based Medicine, Lagos, Nigeria; 4 Kintampo Health Research Centre, Ghana Health Service, Kintampo, Ghana; 5 Department of Clinical Psychology, School of Health in Social Science, Medical School, The University of Edinburgh, United Kingdom; 6 Edinburgh Dementia Prevention, Centre for Clinical Brain Sciences, University of Edinburgh, United Kingdom; 7 King Edward Memorial Hospital Research Centre (KEMHRC), Pune, India; 8 Usher Institute, University of Edinburgh, United Kingdom; 9 Global Public Health, Jeffrey Cheah School of Medicine and Health Sciences, Monash University Malaysia; 10 South East Asia Community Observatory, Monash University Malaysia, Malaysia; 11 Departamento de Neurologia, Faculdade de Medicina da Universidade de São Paulo, São Paulo (SP), Brazil; 12 Tehran University of Medical Sciences (TUMS), Tehran, Iran; 13 School of Psychological Sciences and Health, University of Strathclyde, Glasgow, United Kingdom; 14 Alzheimer’s Scotland Dementia Research Centre, Edinburgh University, United Kingdom; 15 Autonomous University of the Caribbean, Barranquilla, Colombia; 16 Department of Medicine, George Washington School of Medicine & Health Sciences, Washington, DC, United States; 17 Health System and Population Studies Division, icddr,b Bangladesh; 18 Institute of Mental Health, Division of Psychiatry and Applied Psychology, School of Medicine, Nottingham University, United Kingdom; 19 Dementia Care & Research Centre, Peking University Institute of Mental Health (Sixth Hospital), Beijing, China; 20 Beijing Dementia Key Lab, Beijing, China; 21 National Clinical Research Centre for Mental Disorders, Key Laboratory for Mental Health, Beijing, China; 22 Department of Psychology, Faculty of Health and Life Sciences, Northumbria University, Newcastle upon Tyne, United Kingdom; 23 Edinburgh – Centre for Research on the Experience of Dementia, School of Health in Social Science, University of Edinburgh, Edinburgh, United Kingdom; 24 Departamento de Clínica Médica, Faculdade de Medicina da Universidade Federal de Minas Gerais, Belo Horizonte (MG), Brazil

Dementia is a costly neurodegenerative disease that affects 47 million people worldwide. This figure is predicted to rise to 75 million by 2030 and 132 million by 2050 [1]. Currently, the majority of people (approximately 63%) living with dementia are in low- and middle-income countries (LMICs) and due to their large rapidly ageing populations, this number is going to continue to grow. For example, the *Global Health Epidemiology Reference Group (GHERG)* estimated that between 1990 and 2010, China had experienced a 2.5-fold increase in the number of dementia cases, increasing the number of people living with dementia in China alone to 9.19 million, approximately one-fifth of the global dementia burden in 2010 [2].

Dementia affects not only people with the disease, but it also places tremendous health and financial pressure on families and other caregivers, as well as on the health and social care systems. The global cost of dementia is currently estimated at USD 800 billion per annum, and this is predicted to rise to USD 2 trillion by 2030 [3]. Unless effective action is taken, dementia will have significant detrimental personal, health, and socio-economic impacts worldwide. With no disease modifying treatments available, the World Health Organisation’s (WHO) Global Action Plan on the Public Health Response to Dementia emphasises developing effective, low cost, and contextually appropriate strategies for reducing risk and delaying or preventing dementia onset and progression. Such strategies have become top priorities in the global dementia agenda [4]. Another priority is the creation of dementia friendly communities, vital to ensuring that people with cognitive impairment and dementia do not become isolated.

## DEMENTIA AND RESEARCH PRIORITIES

To align research investment to the above agenda, in 2015, several of our authors assisted the WHO in a study that mobilised experts and stakeholders from a wide range of disciplines and sectors (including funders, policy makers, professional, and civil societies) in 39 countries to set health research priorities to reduce the global burden of dementia by 2025 [5]. While the top research priorities focused on prevention, risk reduction, and on the delivery and quality of care for people with dementia and their caregivers, other priorities also included research in the development of cross-cultural diagnosis, biomarkers, and treatment, as well as improving public awareness and understanding of dementia. The study highlighted the importance of interdisciplinary and intersectoral collaboration to motivate investment to support critical research priorities that reduce disease burden, and enable the knowledge generated from the research to have real-life impact through knowledge exchange and specific actions.

To develop the identified research priorities into meaningful research, colleagues from the Centre for Global Health and Centre for Medical Informatics (Usher Institute), Edinburgh Dementia Prevention (Edinburgh Neuroscience), and Centre for Research on the Experience of Dementia came together and asked how new developments in our respective fields could be aligned to the research priorities generated by the WHO-led study. In 2018, we invited colleagues from nine LMICs – Bangladesh, Brazil, China, Ghana, India, Iran, Malaysia, Nigeria, Uruguay – to Edinburgh to share with us the state of epidemic preparedness and research needs in their countries, and explore ways in which new developments in our respective fields can be shared to benefit LMIC settings.

The Global Dementia Prevention Program (GloDePP) was formed as a platform to facilitate research around the key priorities identified. In 2019, the group was further expanded to include colleagues from the University of Strathclyde, and the University of Nottingham. We also developed an alliance with the Latin America and Caribbean Consortium on Dementia (LAC-CD).

## OUR VISION

GloDePP envisages a world in which those living in the poorest countries have access to world class, culturally sensitive, cost appropriate diagnostics, prevention, management and treatment for dementia.

## OUR MISSION

Taking a multidisciplinary and multisectoral approach to address some of the key challenges posed by dementia, we seek to create a global network of researchers with expertise in clinical sciences, artificial intelligence (AI), digital technologies, neuroscience, data, health, and social sciences to conduct collaborative research to mitigate the impact of dementia in the world’s poorest countries.

## OUR STRATEGIC GOALS

Our strategic goals are to:

- Develop contextually appropriate tools and conduct research to assess early and mid-life risk exposures to dementia, gaps in policy and health services delivery, and public sentiments toward dementia;
- Develop and apply novel, low-cost methods for analysing biomarkers of brain changes in pre-clinical populations across different language and cultural groups;
- Develop and pilot contextually appropriate m-health, e-health, and AI tools for early dementia prevention, detection, and intervention;
- Generate information to identify investment priorities on dementia;
- Strengthen the evidence base for integrating the experience of people living with cognitive impairment and dementia, as well as caregivers, into public policy;
- Develop a “global data science platform” to enable data sharing from diverse sources that could be used to study dementia in greater detail especially focussing on cognitive decline across different groups and contexts globally;
- Develop public health approaches for dementia risk reduction and prevention, and “personalised preventive medicine” at low cost;
- Identify policies and strategies to promote healthy brain ageing and create dementia friendly environments; and,
- Develop global, generic guidelines for equitable access to dementia prevention and management (and future cures) that are contextually adaptable across geographies and cultures

## *JOGH-JOGHR* THEME ISSUE ON GLOBAL DEMENTIA

To mark the beginning of the GloDePP collaboration, we developed this theme issue on global dementia. The theme issue consists of 14 papers published over 3 issues of the *Journal of Global Health (JoGH)* and its sister journal, the *Journal of Global Health Reports (JoGHR*) (http://joghr.org). The papers are divided into two sub-themes: **(**i) those that document some of the common needs and challenges in dementia prevention and epidemic preparedness in low-resource settings; and (ii) papers that illustrate new research advances (especially in neuropsychology, mobile technology, and data sciences) and locally generated low-cost solutions in LMICs that could offer collaborative ways forward.

Under sub-theme 1, Adeloye et al. [6], Nitrini et al. [7], Noroozian et al. [8], Nulkar et al. [9], Nyame et al. [10], Poon et al. [11], Wang et al. [12] describe the rising trend of dementia in China, Ghana, Nigeria, India, Iran, Latin American, and Southeast Asian countries, the paucity of epidemiological and other research into dementia, and the fragmentation of diagnosis, prevention, and care services in these settings leading to large gaps in diagnosis (up to 90%), treatment, and support services resulting in over-reliance on informal care by the family. Scarcity of funding aside, the lack of recognition towards dementia by governments and health authorities is seen by many of our authors as a key contributor to the paucity of research and delivery of necessary social and health services [7-10,12]. There is currently an absence of national dementia strategies across these LMICs. Further, the lack of a culturally valid assessment tool for cognitive impairment, and the high cost of diagnostic imaging tools such as fluorodeoxyglucose (FDG)-positron emission tomography (PET) and Pittsburgh Compound-B (PIB) PET, are other challenges for research and diagnosis in LMICs [13-14]. Calia et al. [13] explore the differences in the representation of dementia between people from the United States, United Kingdom, and China. They caution readers about the effect that these differences might have on the ways in which dementia is experienced and care is provided. Calia et al. [13], Nitrini et al. [7], Parra et al. [14], Wang et al. [15], and Watermeyer & Calia [16] discuss the cultural, educational, and linguistic biases in commonly used neuropsychological tools that are used for detecting cognitive impairments. They highlight the effect these issues have on research opportunities and clinical practice in non-English speaking and illiterate populations, especially in LMICs.

Under sub-theme 2 (low-cost and efficient solutions), Watermeyer & Calia [16], Parra et al. [14], Muniz-Terrera et al. [17], and Danso et al. [18] offer exciting insights into how new advances in neuropsychology, mobile technology, and data sciences (eg, speech and text analysis) can overcome language, educational, and cultural biases as well as problems with repeated measures embedded within established cognitive measures. These emerging tests and technologies also improve precision and sensitivity in the detection of pre-symptomatic Alzheimer’s disease. These advances offer “a new paradigm for cognitive testing” that opens up possibilities for the creation of harmonised longitudinal data on cognitive ageing across diverse populations globally [17]. As many of these emerging tools and technologies are designed with affordability and ease of administration in mind, they offer important opportunities for disease prevention as well as bridging the gap in knowledge generation between high income countries and their LMIC counterparts. Over time, the shift in research from symptomatic to pre-symptomatic Alzheimer’s disease offers new possibilities for prevention worldwide.

As an immediate solution, making use of established neuropsychological tools for detecting cognitive impairments, Wang et al. [15] describe a process for selecting and creating normative values for a set of established neuropsychological tests for measuring mild cognitive impairment (MCI) and Alzheimer’s disease in the *Chinese Neuropsychological Normative Project (CN-NORM)*. This has important implications for expanding the epidemiological evidence on dementia and MCI in LMICs. Poon et al. [11] validate estimates of dementia for the Southeast Asian region. The study explores the utility of a simple, free statistical package to generate a normal-normal hierarchical model (NNHM) for disease burden estimates with limited data. The model allows researchers from low-resource settings to engage in disease burden, and estimate modelling.

Finally, to address the fragmentation of services Wang et al. [12] describe a model of continuum of care currently being implemented in China. Based on the WHO mhGAP guideline, the model aims to cut costs by mobilising and training community and primary care workers for screening and case management, capping the investment in specialist and institutional based resources. Similarly, as an interim low-cost solution for bridging the gaps in dementia training for clinical, health, and social workers, Noroozian et al. [8] describe the memory clinic initiative (MCI) developed in Iran, which may be used as a model for other LMIC settings.

We are excited by the initiative and hope that the articles in this theme issue will inspire advances in collaborative research that might help strengthen knowledge around the epidemiology of dementia in LMICs, strategies for screening and diagnosis of pre-symptomatic dementia, training of the social and health care work-force, research capacity building, and data analysis. We envisage that the GloDePP collaboration will support the global response to the dementia challenge. We welcome new collaborators.
